# Which Zirconia Surface-cleaning Strategy Improves Adhesion of Resin Composite Cement after Saliva Contamination? A Systematic Review and Meta-Analysis

**DOI:** 10.3290/j.jad.b2916437

**Published:** 2022-04-13

**Authors:** Nathalia Ramos da Silva, Gabriela Monteiro de Araújo, Taciana Emília Leite Vila-Nova, Marcela Guedes Pereira Gouvêa Bezerra, Patrícia dos Santos Calderon, Mutlu Özcan, Rodrigo Othávio de Assunção e Souza

**Affiliations:** a PhD Student, Federal University of Rio Grande do Norte (UFRN), Department of Dentistry, Natal, RN, Brazil. Wrote the manuscript, consulted on and performed statistical evaluation, contributed substantially to discussion.; b Dentist in Private Practice, Federal University of Rio Grande do Norte (UFRN), Department of Dentistry, Natal, RN, Brazil. Idea, wrote the manuscript, contributed substantially to discussion.; c PhD Student, University of Pernambuco (UPE), School of Dentistry, Recife, PE, Brazil. Idea, wrote the manuscript, contributed substantially to discussion.; d MSc Student, Federal University of Rio Grande do Norte (UFRN), Department of Dentistry, Natal, RN, Brazil. Wrote the manuscript, consulted on and performed statistical evaluation.; e Adjunct Professor, Federal University of Rio Grande do Norte (UFRN), Department of Dentistry, Natal, RN, Brazil. Consulted on and performed statistical evaluation, proofread the manuscript.; f Professor, University of Zurich, Center of Dental Medicine, Division of Dental Biomaterials, Clinic for Reconstructive Dentistry, Zurich, Switzerland. Idea, contributed to discussion, proofread the manuscript.; g Adjunct Professor, Federal University of Rio Grande do Norte (UFRN), Department of Dentistry, Natal, RN, Brazil. Idea, hypothesis, proofread the manuscript.

**Keywords:** adhesion, bond strength, decontamination, dental materials, saliva contamination, surface cleaning, zirconia

## Abstract

**Purpose:**

To identify the most effective cleaning method for saliva-contaminated zirconia surface before adhesive cementation through a systematic review and meta-analysis.

**Materials and Methods:**

PubMed, Scopus, and Web of Science databases were searched to select in vitro studies published through October 2021. Studies that did not perform aging methods, had a sample size less than 5 per group, or did not present a group with zirconia contaminated only with saliva were excluded. Data were extracted and risk of bias was assessed. Statistical analysis comparing the cleaning methods was conducted, and the standardized mean difference was assessed using the R software program.

**Results:**

Among 804 potentially eligible studies, 36 were selected for full-text reading, of which 13 were included in qualitative analysis, and 11 of these were subsequently included in the quantitative analysis. A meta-analysis revealed a significant difference in the bond strength between the cleaning methods. Sandblasting with Al_2_O_3_ showed a higher bond strength than cleaning solution (Ivoclean, Ivoclar Vivadent) (p < 0.01, I^2^ = 65%), and both methods promoted higher resin-bond strength to zirconia than water cleaning. In addition, there was no significant difference in the bond strength between alcohol (p = 0.35, I^2^ = 79%), phosphoric acid (p < 0.23, I^2^ = 90%), and water cleaning.

**Conclusion:**

Sandblasting with Al_2_O_3_ seems to be the best method for zirconia surface cleaning before adhesive luting, promoting better resin-bond strength to zirconia.

Metal-free ceramic restorations are increasingly used in dentistry due to the greater demand for cosmetic treatments. Zirconia ceramics stand out as the material of choice because of their excellent mechanical properties, such as high flexural strength (> 900 MPa),^39^ in addition to esthetic properties, with the recent introduction of translucent and ultratranslucent zirconia on the market.^46^ Thus, zirconia ceramics have expanded their indication to monolithic zirconia crowns, inlays, onlays, and veneers, which require effective adhesive cementation.^37^ However, zirconia is a crystalline ceramic and conventional surface treatment with hydrofluoric acid followed by silane application is not effective, as opposed to silica-based ceramics.

Therefore, zirconia cementation is a critical stage in this type of treatment,^45,49^ and any improvement in this interface is important for the clinical durability of these restorations. Clinical studies report that fractures in ceramic crowns^40,47^ and adhesion failure between zirconia ceramics and resin composite cement compromise the longevity of treatments.^29,35,38,40^ One of the factors related to adhesive failures is contamination by saliva, blood, or silicone during the try-in or cementation of the zirconia restoration,^3,53^ which reduces the contact between cement and zirconia, compromising adhesion. Saliva contains phosphate groups, with which zirconia has a great affinity.^19^ Thus, it is necessary to remove them so they cannot influence adhesion.

Previous studies^10,34^ have shown that salivary contaminants can decrease the resin bond strength to zirconia. Thus, several cleaning methods of contaminated zirconia surfaces have been investigated, including cleaning with water,^14^ phosphoric acid,^15^ alcohol,^10,33^ aluminum oxide (Al_2_O_3_) sandblasting,^27,54^ plasma,^34^ sodium hypochlorite,^15^ and cleaning solution (Ivoclean, Ivoclar Vivadent; Schaan, Liechtenstein).^8^ The literature reports several results regarding the effect of cleaning methods on the bond strength of zirconia to resin composite cement.^8,10,14,15,27,33,34,42^ Cleaning saliva-contaminated zirconia with water or alcohol does not seem effective in removing the organic coating formed by saliva contamination.^34^ On the other hand, mechanical cleaning with Al_2_O_3_ sandblasting, plasma, and cleaning solutions have shown promising results.^10,34,54^

However, consensus is lacking on the most effective method for cleaning zirconia restorations contaminated with saliva during the try-in step. Moreover, there is no systematic review or meta-analysis regarding this in the scientific literature. Thus, this systematic review and meta-analysis aimed to evaluate the effect of cleaning methods for saliva-contaminated zirconia on the bond strength to composite cement. The null hypothesis was that bond strength between zirconia and composite cement was not influenced by the cleaning methods.

## Materials and Methods

This systematic review was performed according to the Preferred Reporting Items for Systematic Reviews and Meta-Analyses (PRISMA) statement^24^ and the population, intervention, comparison, outcomes, and study design (PICOS) approach. In this review, the PICOS were defined as follows: population: saliva-contaminated zirconia; intervention: surface cleaning methods; comparison: alcohol, cleaning solution (Ivoclean, Ivoclar Vivadent), phosphoric acid, Al_2_O_3_ sandblasting, and water; outcome: bond strength; study design: in vitro studies. The research question was: What is the most effective cleaning method for saliva-contaminated zirconia surfaces to achieve the best bond strength to composite cement?

### Search Strategy

The search was carried out in PubMed and adapted to Scopus and Web of Science electronic databases in May 2021, updated in October 2021. The search strategy was elaborated for PubMed based on the concepts of populations, intervention, and outcome of the PICO question. The search strategy was composed of the MeSH (Medical Subject Headings) terms and the free terms related to the concepts. Within each concept, MeSH and free terms were combined using the Boolean operator OR. Next, the results of the concepts were combined using the Boolean operator AND. The search strategy was adapted to each electronic database (Table 1).

**Table 1:** Search strategy in each electronic database

Database	Search strategy
PubMed	(“zirconia”[Title/Abstract] OR “Y-TZP”[Title/Abstract] OR “zirconium”[Title/Abstract]) AND (“decontamination”[MeSH Terms] OR “decontamination”[Title/Abstract] OR “decontaminated”[Title/Abstract] OR “contamination”[Title/Abstract] OR “contaminations”[Title/Abstract] OR “contaminated”[Title/Abstract] OR “cleaning”[Title/Abstract] OR “cleaned”[Title/Abstract] OR “saliva”[MeSH Terms] OR “saliva”[Title/Abstract] OR “salivary”[Title/Abstract]) AND (“bond”[Title/Abstract] OR “bond strength”[Title/Abstract] OR “bonding”[Title/Abstract] OR “adhesion”[Title/Abstract])
Scopus	TITLE-ABS-KEY (“zirconia” OR “Y-TZP” OR “zirconium”) AND TITLE-ABS-KEY ( “decontamination” OR “decontaminated” OR “contamination” OR “contaminations” OR “contaminated” OR “cleaning” OR “cleaned” OR “saliva” OR “salivary”) AND TITLE-ABS-KEY (“bond” OR “bond strength” OR “bonding” OR “adhesion”)
Web of Science	TS=(“zirconia” OR “Y-TZP” OR “zirconium”) AND TS=( “decontamination” OR “decontaminated” OR “contamination” OR “contaminations” OR “contaminated” OR “cleaning” OR “cleaned” OR “saliva” OR “salivary”) AND TS=(“bond” OR “bond strength” OR “bonding” OR “adhesion”)

### Eligibility Criteria

The inclusion criteria for selection were in vitro studies published up to October 2021 that assessed the effect of cleaning strategies for contaminated zirconia on bond strength to composite cement; studies that included groups of zirconia samples only contaminated with saliva before the application of cleaning methods; studies that performed at least one method of aging the samples after cementation; studies that performed tensile or shear bond strength testing, and studies in which the adhesive interface tested was zirconia/composite cement or zirconia/composite cement/resin composite. The exclusion criteria were studies with a sample size less than 5 in each group; studies that only presented groups in which the zirconia surface was not contaminated before applying the cleaning method; studies that did not submit the samples to aging methods or that performed storage for less than 30 days or thermocycling for less than 5000 cycles, and studies that evaluated only one cleaning method.

### Study Selection and Data Collection

The studies found in the search were imported into Endnote X9 software (Thomson Reuters; Toronto, Canada) for the removal of duplicates. Then, the references were imported to Ryyan (https://www.rayyan.ai) for study selection. Screening and selection were independently assessed by 2 researchers (N.R.S. and M.G.P.G.B.) in two phases. In the first phase, after exclusion of duplicated studies, titles and abstracts were assessed according to the eligibility criteria to select relevant studies for full-text reading. In the second phase, full-text reading of the selected studies was also performed independently by 2 researchers (N.R.S. and M.G.P.G.B) according to the eligibility criteria. The kappa test was performed to assess the agreement among the researchers in the study selection in the first and second phases. Disagreements in selections were resolved with the help of a third author (G.M.A.). The reference lists of the articles were manually searched for relevant studies.

### Data Collection

Data of the included studies were extracted independently by 2 researchers (N.R.S. and M.G.P.G.B). The data were collected in a specific form created in Microsoft Office Excel 2016 (Microsoft; Redmond, WA, USA) to extract the information: author and year, sample size per group, zirconia brand, contamination agent, cleaning methods, bonding procedure, aging, bond strength test, and results. An attempt to contact the corresponding author was performed for studies with missing or unclear data information.

### Risk of Bias

The risk of bias of the included studies was independently assessed by 2 researchers (N.R.S. and M.G.P.G.B). The parameters for risk of bias assessment were adapted from previous studies:^25,26,32,43^ sample size calculation; specimen randomization; clearly described, standardized, and reproducible specimen preparation; clearly specified aging parameters; specimen preparation and test execution following the International Organization for Standardization (ISO); single operator applied the protocol, bond strength tests performed by a blinded operator; and failure mode evaluation. Values from 0 to 2 were assigned for each parameter: 0: the study clearly reported the parameter; 1: the parameter was reported, but its precise execution was not; 2: the parameter was not reported at all. The scores were then added, and if the total ranged from 0 to 4, the study was considered low risk of bias; from 5 to 9, medium risk; and from 10 to 14, high risk of bias. Studies with a high risk of bias were excluded.

### Meta-Analysis

The outcomes extracted for quantitative analysis were mean bond strength after aging in MPa, standard deviation, and number of specimens per group. These data were converted to SD in studies missing standard deviations (SD), but which reported the interquartile ranges or confidence interval (CI).^12^ Each group was considered independently and identified by letters if the study had two or more experimental groups.

Five analyses were conducted to compare the effect of cleaning techniques on bond strength: alcohol vs water (control); cleaning solution (Ivoclean, Ivoclar Vivadent) vs water; phosphoric acid vs water; sandblasting vs water; and cleaning solution vs sandblasting. Studies which presented groups with zirconia contaminated by saliva and applied the cleaning methods of one of the five comparisons were included in the meta-analysis. However, the standardized mean difference (SMD) was used, since studies measure bond strength by different methods (shear bond strength and micro- or macrotensile bond strength). The SMD and 95% confidence interval (CI) were calculated and p-values less than 0.05 were considered to be statistically significant in all comparisons. A random-effect model was used for all of the meta-analysis. Heterogeneity was assessed with the I^2^ statistic and classified as low (I^2^≤25%), moderate (I^2^≤50%), and high (I^2^>75%). The R version 4.0.3 (The R Foundation for Statistical Computing [http://www.r-projetct.org/]) was used for the meta-analysis.

## Results

**Fig 1:**
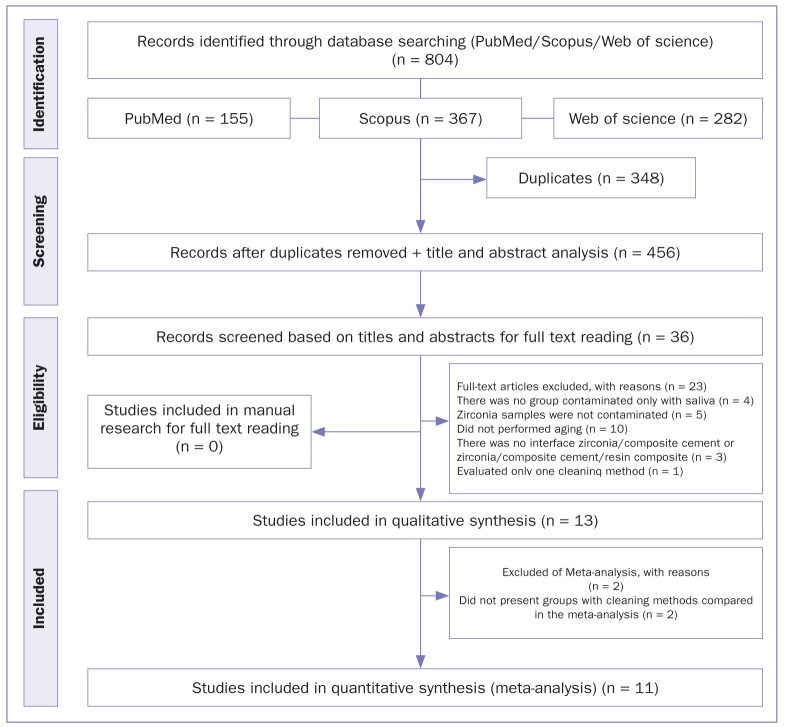
Flowchart of the study selection process.

A total of 804 studies were identified by searching the three electronic databases (PubMed, Scopus, Web of Science). After excluding duplicates in the first phase, 456 titles and abstracts were analyzed based on eligibility criteria, and 36 records were selected for full-text reading (second phase). Among these, thirteen studies^5,8,10,14,15,17,21,22,34,41,52,54,55^ were included in the qualitative analysis, and 23 studies were excluded after the second phase (Table 2). No studies from the manual search were included. Of these 13 included studies, 11 were included in the meta-analysis.^5,8,14,15,17, 21,22,34,52,54,55^ Two studies were excluded from the meta-analysis because they did not present groups cleaned with alcohol, cleaning solution, phosphoric acid, or sandblasting and water (Fig 1). A kappa test value of 0.78 (substantial agreement) was obtained in the first phase, with 0.84 in the second phase (almost perfect).^20^

**Table 2:** Description of the excluded studies and reasons for exclusion

Authors	Title	Reason for exclusion
Al-Dobaei E, Al-Akhali M, Polonskyi O, Strunskus T, Wille S, Kern M.	Influence of cleaning methods on resin bonding to contaminated translucent 3Y-TZP ceramic. J Adhes Dent 2020;22:383-391.	There were no groups contaminated only with saliva
Quaas AC, Yang B, Kern M	Panavia F 2.0 bonding to contaminated zirconia ceramic after different cleaning procedures. Dent Mater 2007;23:506-512.	
Yang B, Scharnberg M, Wolfart S, Quaas AC, Ludwig K, Adelung R, Kern M.	Influence of contamination on bonding to zirconia ceramic. J Biomed Mater Res B Appl Biomater 2007;81:283-290	
Phark JH, Duarte S Jr, Kahn H, Blatz MB, Sadan A.	Influence of contamination and cleaning on bond strength to modified zirconia. Dent Mater 2009;25:1541-1550	
Attia A, Kern M.	Effect of cleaning methods after reduced-pressure air abrasion on bonding to zirconia ceramic. J Adhes Dent 2011;13:561-567.	Zirconia specimenss were not contaminated
Attia A, Lehmann F, Kern M.	Influence of surface conditioning and cleaning methods on resin bonding to zirconia ceramic. Dent Mater 2011;27:207-213.	
Canullo L, Micarelli C, Bettazzoni L, Magnelli A, Baldissara P.	Shear bond strength of veneering porcelain to zirconia after argon plasma treatment. Int J Prosthodont 2014;27:137-139.	
Negreiros WM, Ambrosano GMB, Giannini M.	Effect of cleaning agent, primer application and their combination on the bond strength of a resin cement to two yttrium-tetragonal zirconia polycrystal zirconia ceramics. Eur J Dent 2017;11:6-11.	
Lümkemann N, Schönhoff LM, Buser R, Stawarczyk B.	Effect of cleaning protocol on bond strength between resin composite cement and three different CAD/CAM Materials. Materials (Basel) 2020;13:4150.	
Aladağ A, Elter B, Çömlekoğlu E, Kanat B, Sonugelen M, Kesercioğlu A, Özcan M.	Effect of different cleaning regimens on the adhesion of resin to saliva-contaminated ceramics. J Prosthodont 2015;24:136-145.	No aging was performed
Atoche-Socola KJ, Arriola-Guillén LE, López-Flores AI, Garcia IM, Huertas-Mogollón G, Collares FM, Branco Leitune VC.	Microshear bond strength of dual-cure resin cement in zirconia after different cleaning techniques: an in vitro study. J Adv Prosthodont 2021;13:237-245.	
Pak Tunc E, Chebib N, Sen D, Zandparsa R.	Effectiveness of different surface cleaning methods on the shear bond strength of resin cement to contaminated zirconia: an in vitro study. J Adhes Sci Technol 2015:30:1-12.	
Sankar S, Kondas VV, Dhanasekaran SV, Elavarasu PK.	Comparative evaluation of shear bond strength of zirconia restorations cleansed various cleansing protocols bonded with two different resin cements: An In vitro study. Indian J Dent Res 2017;28:325-329.	
Irmak Ö, Yaman BC, Orhan EO, Kılıçarslan MA, Mante FK, Ozer F.	Influence of cleaning methods on bond strength to saliva contaminated zirconia. J Esthet Restor Dent 2018;30:551-556.	
Takahashi A, Takagaki T, Wada T, Uo M, Nikaido T, Tagami J.	The effect of different cleaning agents on saliva contamination for bonding performance of zirconia ceramics. Dent Mater J 2018;37:734-739.	
Wattanasirmkit K, Charasseangpaisarn T.	Effect of different cleansing agents and adhesive resins on bond strength of contaminated zirconia. J Prosthodont Res 2019;63:271-276.	
Zhang J, Hu W, Stijacic T, Chung K, Li T, Shen Z	Bonding of novel self-glazed zirconia dental ceramics. Adv Appl Ceram 2019;118:37-45.	
Joukhadar C, Osman E, Rayyan M, Shrebaty M	Comparison between different surface treatment methods on shear bond strength of zirconia (in vitro study). J Clin Exp Dent 2020;12:e264-e270	
Noronha MDS, Fronza BM, André CB, de Castro EF, Soto-Montero J, Price RB, Giannini M.	Effect of zirconia decontamination protocols on bond strength and surface wettability. J Esthet Restor Dent. 2020;3:521-529.	
Angkasith P, Burgess JO, Bottino MC, Lawson NC.	Cleaning methods for zirconia following salivary contamination. J Prosthodont 2016;25:375-379.	There was no interface zirconia/composite cement or zirconia/composite cement/resin composite
Koko M, Takagaki T, Abdou A, Wada T, Nikaido T, Tagami J.	Influence of 10-methacryloyloxydecyl dihydrogen phosphate (MDP) incorporated experimental cleaners on the bonding performance of saliva-contaminated zirconia ceramic. Clin Oral Investig 2022;26:1785-1795.	
Krifka S, Preis V, Rosentritt M.	Effect of decontamination and cleaning on the shear bond strength of high translucency zirconia. Dent J (Basel) 2017;5:32.	
Rui L, Ma SQ, Liu ZH, Chen ML, Liu J, Wu J, Wang C, Liu Z, Guo ZG, Lu RJ.	High shear bond strength between zirconia ceramic and resin cement via surface treatment and cleaning. Mater Res Express 2021;8:105402.	Evaluated only one cleaning method

Most of the studies included in the systematic review had a medium risk of bias (score between 5 and 9) and sample calculation bias, operator and blinding-related bias, and standardization bias. Only the study by Mangione and Özcan^21^ was classified as low risk of bias (Table 3). The characteristics of the included studies are presented in Table 4. The studies used yttria-stabilized tetragonal zirconia (Y-TZP) from different manufacturers. Most of the studies applied Al_2_O_3_ sandblasting before contamination. The studies applied different bonding protocols such as composite cement with 10-methacryloyloxy-decyl-dihydrogenphosphate (10-MDP),^10,15,21,22,34,41,52,54,55^ ceramic primer with 10-MDP and composite cement,^5,8,14,17^ and conventional composite cement.^5,41^ The aging methods varied from 5000 thermocycles to 150 days of water storage associated with 37,500 thermocycles. Half of the studies performed shear bond strength testing,^5,8,14,15,21,22,54^ and the other half performed tensile bond strength testing.^10,17,34,41,52,55^

**Table 3:** Risk of bias in the included studies for qualitative analysis.

Author/Year	Sample size	Randomization	Specimens preparation	Standardization of procedures	Operator	Blinding	Failure Analysis	Risk of bias
Attia and Ebeid, 20205	0	2	0	1	2	2	0	7
Feitosa et al, 20158	2	1	0	1	0	2	0	6
Güers et al, 201910	2	1	0	1	2	2	0	8
Ishii et al, 201514	2	1	1	1	2	2	0	9
Kim et al, 201515	2	2	0	1	2	2	0	9
Klosa et al, 201417	2	2	0	1	2	2	0	9
Mangione and Özcan, 201921	0	1	0	1	0	2	0	4
Martínez-Rus et al, 202122	0	0	0	1	2	2	0	5
Piest et al, 201834	2	1	0	1	2	2	0	8
Samran et al, 201941	2	2	0	1	2	2	0	9
Yang et al, 200852	2	2	0	1	2	2	0	9
Yoshida, 201854	2	1	0	0	2	2	0	5
Zhang et al, 201055	2	2	0	1	2	2	0	9

0: the parameter is clearly described; 1: the parameter is reported but the precise execution is not clear; 2: the parameter is not specified or is not reported. Total sum between 0 and 4: low risk; between 5 and 9: medium risk; and between 10 and 14: high risk of bias.

**Table 4:** Characteristics of the included studies

Authors, year	N/zirconia, manufacturer	Contaminants	Cleaning methods	Bonding procedure/composite cement	Storage/ aging	Bond strength test	Results after aging (p < 0.05)
Attia and Ebeid, 2020^5^	10/ Katana Zirconia STML, Kuraray Noritake)	Saliva	With contamination: Water (W): 15 s 70% isopropanol (AL): 120 s Zirclean (Bisco) (ZC): 20 s 5.25% sodium hypochlorite (NaOCl): 120 s *SB before contamination	Composite cement cylinders bonded to ceramic RelyX Unicem (3M Oral Care)	TC 5000	SBS	AL = ZC = NaOCl > W
Feitosa et al, 2015^8^	12/Diazir Full- Contour, Ivoclar Vivadent	Saliva	Control (C): without contamination With contamination: Water (W): 15 s 37% phosphoric acid (PA): 60 s Ivoclean (IC): 20 s 70% isopropanol (AL): 120 s *SB before contamination	Composite cement cylinders bonded to ceramic Monobond Plus + Multilink Automix (Ivoclar Vivadent)	150 days or TC 5000	SBS	150 days: C = W = IC = AL; C = W = IC > PA; AL = PA TC 5000: C = IC = W; C = IC >AL = PA W > PA
Güers et al, 2019^10^	8/ICE Zirkon Translucent, Zirkonzahn	Saliva or silicone disclosing agent	Control (C): without contamination With contamination saliva or silicone Ultrasonic bath with 99% isopropanol (UAL): 180 s UAL + plasma air gas (air): 15 min UAL + plasma 1:1 argon-oxygen (AO) gas: 15 min UAL + ultrasonic bath with enzymatic detergent (Sekusept Multienzyme P) (ED): 10 min *SB before contamination	Resin composite cylinders (Clearfil DC Core New Bond, Kuraray Noritake) bonded to ceramic with composite cement Panavia 21 TC (Kuraray Noritake)	150 days + TC 37,500	TBS	Saliva contamination: Air = AO > ED > UAL Silicone contamination: Air = AO = UAL > ED
Ishii et al, 2015^14^	10/IPS e.max ZirCAD, Ivoclar Vivadent	Saliva	Control (C): without contamination With contamination: Water (W): 30 s 37% phosphoric acid (PA): 30 s Ivoclean (IC): 20 s Al2O3 sandblasting (SB): 20 s *SB before contamination	Composite cement cylinders bonded to ceramic Monobond Plus + Multilink automix (Ivoclar Vivadent)	TC 10,000 or TC 30,000	SBS	10,000 TC: C = SB > IC > PA > W; 30,000 TC: C = SB = IC > PA > W
Kim et al, 2015^15^	12/Lava, 3M Oral Care	Saliva	Control (C): without contamination With contamination: Water (W): 15 s Al2O3 sandblasting (SB): 15 s Ivoclean (IC): 20 s 1%sSodium dodecyl sulfate (SDS): 20 s 1% hydrogen peroxide (HP): 20 s 1% sodium hypochlorite (NaOCl): 20 s *SB before contamination	Composite cement cylinders bonded to ceramic Panavia F 2.0 (Kuraray Noritake)	TC 5000	SBS	C = NaOCl = SB = IC > HP = W = SDS; SDS > HP
Klosa et al, 2014^17^	8/Cerconbase, Degudent	Saliva or silicone disclosing agent	Control (C): without contamination Saliva or silicone contamination: Water (W): 15 s Ultrasonic bath with 99% ethanol (UE): 180 s *SB before contamination	Resin composite cylinders (Multicore Flow, IvoclarVivadent) bonded to ceramic with composite cement Monobond Plus + Multilink Automix (Ivoclar Vivadent)	150 days + TC 37,500	TBS	Saliva and Silicone disclosing C > W = UE
Mangione and Özcan, 2019^21^	10/Metoxid Dental, Thayngen, Switzerland	Saliva	Control (C): without contamination (non-silicatizated) With contamination: *Half of the contaminated samples were previously silica coated (SL) Water (W): 15 s SL + W 37.5% phosphoric acid (PA): 60 s SL + PA	Composite cement cylinders bonded to ceramic Monobond Plus + Variolink II (Ivoclar Vivadent) or Panavia 21 (Kuraray Noritake)	TC 5000	µSBS	Variolink II: SL+W = SL+PA = W = C > PA Panavia 21: SL+W > SL+PA = W; W = C C = PA
Martínez-Rus et al, 2021^22^	15/ Metoxit Z-CAD HD99-10, Metoxit AG	Saliva	Control (C): without contamination With contamination: Water (W): 30 s Ivoclean (IC): 20 s Argon plasma gas (A): 15 min *SB before contamination	Composite cement cylinders bonded to ceramic Panavia SA (Kuraray Noritake)	TC 10,000	SBS	C = IC > A > W
Piest et al, 2018^34^	8/ ICE Zirkon Translucent, Zirkonzahn	Saliva or silicone disclosing agent	Control (C): without contamination With contamination saliva or silicone Water (W): 15 s Ultrasonic bath with 99% isopropanol (UAL): 180 s Plasma air gas (A): 5 min Plasma oxygen gas (O): 5 min Plasma argon gas (Ar) 5 min *SB before contamination	Resin composite cylinders (Clearfil Core New Bond, Kuraray Noritake) bonded with composite cement Panavia 21 TC (Kuraray Noritake)	150 days + TC 37,500	TBS	Saliva contamination: Air = O = A > UAL > W Silicone contamination: UAL > Air = A = O = W
Samran et al, 2019^41^	8/Zenostar Zr Translucent, Wieland Dental	Saliva	Control (C): without contamination Contaminated: Ultrasonic bath with 99% isopropanol (UAL): 180 s Ivoclean (IC): 20 s + UAL *SB before contamination	Resin composite cylinders (MultiCore Flow; Ivoclar Vivadent) bonded to ceramic with composite cement SpeedCem (Icovlar Vivadent); RelyX Unicem (3M Oral Care); Panavia SA (Kuraray Noritake); Bifix SE (VOCO)	150 days + TC 37,500	TSB	SpeedCem and RelyX Unicem: C > IC + UAL > UAL Panavia SA: C = IC+UAL > UAL Bifix SE: C = IC+UAL = UAL (All TSB = zero)
Yang et al, 2008^52^	8/Cercon, DeguDent	Saliva	Control (C): without contamination With contamination: Water (W): 15 s Al_2_O_3_ Sandblasting (SB): 15 s 37% Phosphoric acid (PA): 30 s + 30 s 70% Isopropanol (AL): 120 s *SB before contamination	Resin composite cylinders (Clearfil FII, Kuraray Noritake) bonded to ceramic with composite cement Panavia F 2.0 (Kuraray Noritake)	150 days + TC 37,500	TBS	C=SB > PA > AL=W
Yoshida, 2018^54^	8/Toso Corp	Saliva	Control (C): without contamination With contamination: Water (W): 15 s 40% phosphoric acid (PA): 30 s Ivoclean (IC): 20 s ADG Gel (ADG): 60 s Al_2_O_3_ sandblasting (SB): 15 s *SB before contamination	Resin composite cylinders (Unifil Core EM, GC Corp) bonded to ceramic with composite cement Clearfil Ceramic Primer Plus + Panavia SA Cement Plus (SACP) Handmix or auto-mix Panavia PV5 (Kuraray Noritake)	TC 10,000	SBS	SACP C = ADG = SB > IC > PA > W PV5: C = ADG = SB > IC = PA = W
Zhang et al, 2010^55^	8/Cercon, DeguDent	Saliva or silicone disclosing agent	Control (C): without contamination C + 37% phosphoric acid 37% (C+PA): 30 s With contamination with saliva or silicone: Water (W): 30 s PA: 30 s *Nano-structured alumina coating before contamination	Resin composite cylinders (Clearfil FII, Kuraray Noritake) bonded to ceramic with composite cement Panavia 21 TC (Kuraray Noritake)	150 days + TC 37,500	TBS	C = C+PA Saliva contamination: Control groups = PA > W Silicone contamination: Control groups > PA > W

TC: thermocycling; TSB: tensile bond strength; SBS: shear bond strength.

In order to clean the zirconia surface of salivary contaminants, studies used Al_2_O_3_^14,15,52,54^ or silica-coated alumina particles sandblasting,^21^ cleaning solution,^8,14,15,22,41,54^ phosphoric acid (35% to 40%),^8,14,21,52,54,55^ alcohol (isopropanol or ethanol, immersion or ultrasonic cleaning),^5,8, 10,17,34,41,52^ plasma (air, oxygen, or argon),^10,22,34^ 1%^15^ to 5.23%^5^ sodium hypochlorite, 1% sodium dodecyl sulfate,^15^ 1% hydrogen peroxide,^15^ ADG Gel^54^ (Kuraray Noritake; Osaka, Japan), enzymatic detergent^10^ (Sekusept Multienzyme P, Eco-lab Deutschland; Monheim am Rhein, Germany), and Zirclean^5^ (Bisco; Schaumburg, IL, USA).

**Fig 2:**
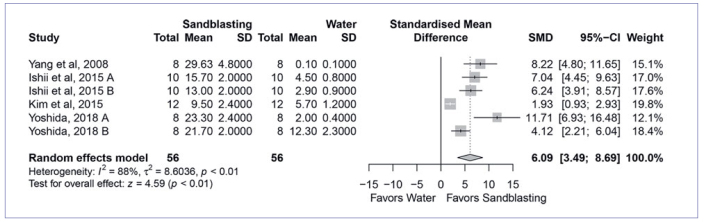
Forest plot summarizing comparison of the “sandblasting” vs “water” cleaning methods. More than one group from the Ishii et al^14^ study (A: 10,000; B: 30,000 thermocycles) entered the meta-analysis. CI: confidence interval; SMD: standardized mean difference.

**Fig 3:**
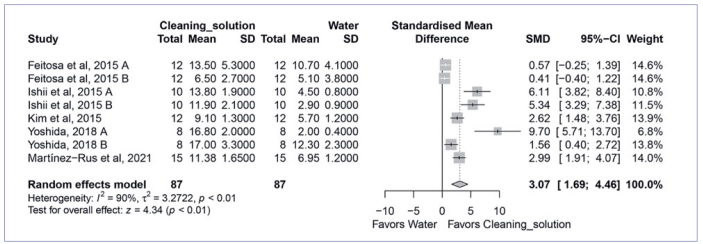
Forest plot summarizing comparison of “cleaning solution” (Ivoclean, Ivoclar Vivadent) vs “water” cleaning methods. More than one group of the studies by Feitosa et al^8^ (A: 5000 thermocycles; B: 150-day water storage), Ishii et al^14^ (A: 10,000; B: 30,000 thermocycles), and Yoshida^54^ (A: handmixed composite cement; B: auto-mix composite cement) entered the meta-analysis. CI: confidence interval; SMD: standardized mean difference.

**Fig 4:**
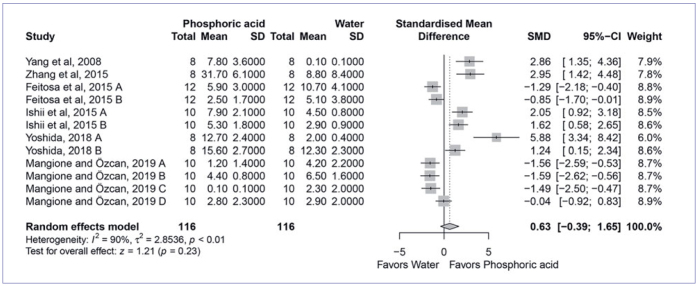
Forest plot summarizing comparison of “phosphoric acid” vs “water” cleaning methods. More than one group of the studies by Feitosa et al^8^ (A: 5000 thermocycles; B: 150-day water storage), Ishii et al^14^ (A: 10,000; B: 30,000 thermocycles), Yoshida^54^ (A: handmixed composite cement; B: auto-mix composite cement), and Mangione and Özcan^21^ (A and B: without and with silicatization, respectively, cemented with 10-MDP-based composite cement; C and D: without and with silicatization, respectively, cemented with methacrylate-based composite cement) entered the meta-analysis. CI: confidence interval; SMD: standardized mean difference.

**Fig 5:**
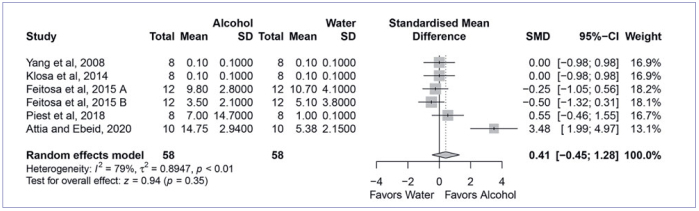
Forest plot summarizing comparison of “alcohol acid” vs “water”. More than one group of the study by Feitosa et al^8^ (A: 5000 thermocycles; B: 150-day water storage), entered in the meta-analysis. CI: confidence interval; SMD: standardized mean difference.

**Fig 6:**
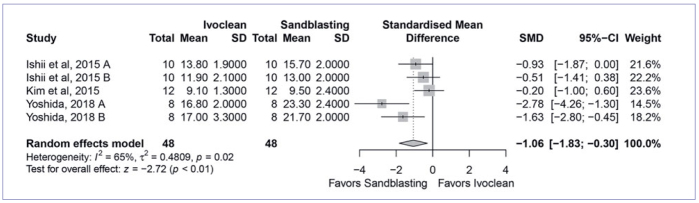
Forest plot summarizing comparison of “cleaning solution” (Ivoclean, Ivoclar Vivadent) vs “sandblasting” cleaning methods. More than one group of the studies of Ishii et al^14^ (A: 10,000; B: 30,000 thermocycles) and Yoshida^54^ (A: handmixed composite cement; B: auto-mix composite cement), entered in the meta-analysis. CI: confidence interval; SMD: standardized mean difference.

Next, the four most used cleaning methods were compared to cleaning with water in the meta-analysis. The quantitative analysis found that cleaning with Al_2_O_3_ sandblasting (SMD, 6.09 [95% CI: 3.49, 8.69], p < 0.01, I^2^ = 88%) (Fig 2) and cleaning solution (SMD, 3.07 [95% CI: 1.69, 4.46], p < 0.01, I_2_ = 90%) (Fig 3) yielded significantly higher bond strength than water cleaning. On the other hand, cleaning with phosphoric acid (SMD, 0.63 [95% CI: -0.39, 1.65], p < 0.23, I^2^ = 90%) (Fig 4) and alcohol (SMD, 0.41 [95% CI: 0.45, 1.28], p < 0.35, I^2^ = 79%) (Fig 5) showed similar bond strengths to water cleaning. A meta-analysis comparing the cleaning solution and Al_2_O_3_ sandblasting was conducted, since they presented the best bond strength results. The quantitative analysis revealed that Al_2_O_3_ sandblasting (SMD, -1.06 [95% CI: -1.83, -0.03], p < 0.01, I^2^ = 65%) mediated significantly higher bond strength than did cleaning solution (Fig 6).

## Discussion

This systematic review and meta-analysis aimed to compare the effect of cleaning methods and indicate which methods present the best results for durable bond strength between zirconia and composite cement. The null hypothesis that the cleaning method for zirconia would not influence the bond strength to composite cement was rejected. The meta-analysis revealed that Al_2_O_3_ sandblasting is the best method for cleaning zirconia samples contaminated with saliva. Moreover, cleaning solution (Ivoclean, Ivoclar Vivadent) is also an excellent alternative, since it presented higher bonding performance than contaminated zirconia samples cleaned with water. However, cleaning with phosphoric acid or alcohol showed similar bond strength to water; thus these methods must be avoided for zirconia cleaning.

The internal surface of an indirect zirconia restoration can be easily contaminated with saliva or other residues during the try-in step. Water cleaning of contaminated zirconia is not an effective method for removing the contaminants. Previous studies have reported that saliva-contaminated zirconia washed with water showed lower bond strength to composite cement than did the control group (non-contaminated zirconia) and other cleaning methods.^5,14,15,22,52,54,55^ Moreover, X-Ray Photoelectron Spectroscopy (XPS) showed that contaminated zirconia samples washed with water present a higher percentage of carbon (C)^15,34,54^ and nitrogen (N),^15,54^ indicating that the residual organic coating formed by saliva contamination remains. Thus, applying a cleaning method other than water is necessary to remove the contaminants from the internal surface of the zirconia, leaving it clean so that the surface treatment can promote surface alterations and improve the adhesion to composite cement.

Al_2_O_3_ sandblasting has been recommended as an alternative for cleaning zirconia restorations contaminated with saliva, blood, and silicone.^30^ The excellent results of this method are due to its ability to mechanically remove saliva elements of zirconia surface, as shown by XPS analysis.^15,54^ Moreover, the bond strength to composite cement achieved by zirconia cleaned with Al_2_O_3_ sandblasting is similar to non-contaminated zirconia.^14,15,52,54^ In most of the included studies, the application protocol used was 50-µm Al_2_O_3_ particles, sandblasted at 2.5-3.0 bars for 15-20 s at a distance of 10 mm.^14,15,52,54^

Although Al_2_O_3_ sandblasting presents excellent bond strength results as a cleaning method, its effect on the mechanical properties of the zirconia is controversial. The impact of sandblasted particles can weaken the zirconia through microcrack formation and surface damage,^11,56^ but when performed under controlled conditions, it may increase the mechanical strength due to the formation of a compressive stress layer.^4^ Thus, the effect of Al_2_O_3_ sandblasting is influenced by the balance between these factors.^13^ In the clinical protocol for cleaning fixed dental prostheses of zirconia, Özcan and Bock^30^ recommended air abrasion from a nozzle distance of 10 mm applied in circling motions to promote uniform surface blasting and reduce the risk of damage to the zirconia surface. However, due to the acquisition cost of a sandblasting device^16^ in addition to the time and work spent on this procedure, many clinicians may delegate this process to the dental laboratory^10^ and search for other methods for cleaning zirconia restorations chairside.

In this context, the cleaning solution (Ivoclean, Ivoclar Vivadent) is also an effective method for cleaning saliva-contaminated zirconia. According to this meta-analysis, the improved bonding performance compared to water cleaning only and showed bond strengths similar to those of the control group of non-contaminated zirconia in most of the studies.^8,14,15,22^ This was confirmed by a recent study^54^ that performed XPS analysis and detected a decrease in molar percentage concentrations of C and N, elements of saliva contamination, in zirconia samples cleaned with cleaning solution. These results may be explained by the mechanism of action of the cleaning solution. This product contains zirconia particles that remove the saliva phosphate groups from the ceramic surface by adsorption.^54^ According to the manufacturer (Ivoclar Vivadent Scientific Documentation, 2011), due to the size and concentration of the zirconium oxide particles in the medium, there is a greater tendency for the phosphate groups of salivary contaminants to bond to the particles from the cleaning solution than to the surface of the zirconia restoration, leaving a clean surface.

In addition, handling this product is simple, which is an advantage. In the included studies, Ivoclean was applied according to the manufacturer’s instructions (Ivoclean was applied on the zirconia surface and allowed to react for 20 s). Al Dobaei et al^2^ reported that application according to the manufacturer’s instructions and performing additional rubbing did not improve the bonding strength of the cleaned zirconia to the composite cement. Thus, the clinical protocol for using the cleaning solution must follow manufacturer’s recommendations.

The results of this meta-analysis indicate that phosphoric acid and alcohol are not satisfactory options for cleaning saliva-contaminated zirconia. Studies^8,50^ have shown that cleaning zirconia ceramics with phosphoric acid is not adequate, since the phosphate groups present in the acid form a layer on the ceramic surface, making it inert to the adhesive and hence compromising the bond strength.^30,53^ Regarding alcohol, most of the included studies used isopropanol for cleaning,^5,8,10,34,41,52^ but ethanol was only used by one study.^17^ The XPS analysis of saliva-contaminated samples ultrasonically cleaned in 99% isopropanol for 3 min showed only a slight decrease in C in comparison to contaminated samples cleaned with water, indicating that this method does not effectively remove the organic coating formed by saliva.^33^ Thus, cleaning saliva-contaminated zirconia surfaces by phosphoric acid or alcohol does not seem to be effective.

Other cleaning methods that have presented promising results were also investigated in the included studies, ie, plasma^10,34^ and sodium hypochlorite.^54^ Plasma can be defined as a fully or partially ionized gas and is considered the fourth state of matter.^10^ It presents antimicrobial effect and is capable to promote surface modifications in contact with different substrates, without causing surface damage.^10^ Due to these properties, plasma has been proposed in several dental fields as dental implants, adhesion, caries, endodontic and periodontal treatment, and tooth bleaching.^7,18^ Moreover, this method can improve the surface energy of zirconia due to the increase of oxygenic polar groups, creating a more hydrophilic surface.^7^ Consequently, the plasma treatment can enhance the adhesion between zirconia and composite cement.^28,51^

The cleaning effect of plasma treatment may be due to the active ions on plasma that are able to break the chemical bond and split large molecule chains into smaller particles.^10^ Plasma treatment based on air,^34^ oxygen,^34^ or argon^22,34^ gas, as well as the combination of ultrasonic isopropanol-cleaning and plasma treatment,^5^ have presented promising results in removing salivary contaminants. With the exception of Martínez-Ruz et al,^22^ the included studies^10,34^ showed that plasma cleaning of saliva-contaminated zirconia promoted similar bond strength to the non-contaminated group. The association between ultrasonic isopropanol cleaning and plasma treatment seems to be an excellent option to mechanically clean mechanical cleaning and chemically condition contaminated zirconia.^10^ Thus, considering several applications of plasma treatment in the dental field and the development of a more user-friendly plasma device,^34,48^ this method may be incorporated into clinical routine in the future.

Another method investigated in the included studies is the use of sodium hypochlorite, which is an antimicrobial and deproteinizing agent.^23,44^ It has been used for cavity^6^ and root disinfection^23^ and to remove organic content from enamel^9^ and dentin^1,6^ substrates to improve the bond strength to resin-based materials. Likewise, its use for zirconia cleaning has shown positive results.^5,15,54^ Sodium hypochlorite used for surface cleaning at 1%^15^ and 5.25%^5^ showed a higher bond strength than water. A cleaning solution with 10% sodium hypochlorite (AD Gel, Kuraray Noritake) used for dentin surface treatment^12^ mediated higher bond strength than water, but similar to Al_2_O_3_ sandblasting and control.^54^ This positive effect of sodium hypochlorite may be related to its capacity to effectively remove the salivary organic material from the zirconia surface.^8,54^ However, as hypochlorite may interfere with resin polymerization,^36^ extensive water rinsing after the application of sodium hypochlorite is recommended to remove the solution residue. Other cleaning methods such as 1% hydrogen peroxide and 1% sodium dodecyl sulfate yielded low bond strengths, similar to water rinsing,^15^ and are thus not indicated for cleaning saliva-contaminated zirconia surfaces.

One of the limitations of this systematic review and meta-analysis is the high heterogeneity of the included studies due to the variability of sample preparation, cementation protocol, aging methods, and bond strength testing. Most of the included studies were classified as medium risk of bias. Information is lacking regarding the sample size calculation, standardized procedures following ISO, standardization of the operator applying the protocols, and blinding of the operator who performed the bond strength test. Thus, the results of this systematic review and meta-analysis must be viewed with caution. Moreover, it was not possible to compare all cleaning methods due to the low number of studies which could be compared.

## Conclusion

Air abrasion with Al_2_O_3_ was shown to be the best method for cleaning zirconia surfaces contaminated with saliva before adhesive luting, promoting better and durable bond strength of resin composite cement to zirconia. Cleaning solution, plasma treatment, and sodium hypochlorite are also satisfactory alternatives for removing salivary contaminants. However, cleaning zirconia surfaces with phosphoric acid or alcohol are not effective methods, and therefore are not recommended for sole usage.

## Clinical relevance:

Considering that zirconia restorations are frequently contaminated by saliva during the try-in steps, air-abrasion with Al_2_O_3_ seems to be the most suitable method for cleaning intaglio surfaces of zirconia restorations prior to surface conditioning and adhesive cementation.
